# Atypical, Composite, or Blended Phenotypes: How Different Molecular Mechanisms Could Associate in Double-Diagnosed Patients

**DOI:** 10.3390/genes13071275

**Published:** 2022-07-19

**Authors:** Erica Rosina, Lidia Pezzani, Laura Pezzoli, Daniela Marchetti, Matteo Bellini, Alba Pilotta, Olga Calabrese, Emanuele Nicastro, Francesco Cirillo, Anna Cereda, Agnese Scatigno, Donatella Milani, Maria Iascone

**Affiliations:** 1 Laboratory of Medical Genetics, ASST Papa Giovanni XXIII, 24127 Bergamo, Italy; erica.rosina@unimi.it (E.R.); lpezzoli@asst-pg23.it (L.P.); dmarchetti@asst-pg23.it (D.M.); m.bellini@asst-pg23.it (M.B.); miascone@asst-pg23.it (M.I.); 2 Paediatric Unit, ASST Papa Giovanni XXIII, 24127 Bergamo, Italy; lpezzani@asst-pg23.it (L.P.); enicastro@asst-pg23.it (E.N.); acereda@asst-pg23.it (A.C.); ascatigno@asst-pg23.it (A.S.); 3 Fondazione IRCCS Ca’ Granda Ospedale Maggiore Policlinico, 20122 Milano, Italy; 4 Auxo-Endocrinology, Diabetology and Medical Genetic Unit, Department of Paediatrics, ASST Spedali Civili, 25123 Brescia, Italy; alba.pilotta@asst-spedalicivili.it; 5 Medical Genetics Unit, Azienda Ospedaliera Universitaria di Modena, 41125 Modena, Italy; calabrese.olga@policlinico.mo.it; 6 Pediatric Hepatology and Paediatric Liver Transplantation, Istituto Mediterraneo per i Trapianti e Terapie ad Alta Specializzazione, 90127 Palermo, Italy; f.cirillo@ismett.edu

**Keywords:** trio-WES, composite phenotype, double diagnosis, deep phenotyping

## Abstract

In the last few years, trio-Whole Exome Sequencing (WES) analysis has revolutionized the diagnostic process for patients with rare genetic syndromes, demonstrating its potential even in non-specific clinical pictures and in atypical presentations of known diseases. Multiple disorders in a single patient have been estimated to occur in approximately 2–7.5% of diagnosed cases, with higher frequency in consanguineous families. Here, we report the clinical and molecular characterisation of eight illustrative patients for whom trio-WES allowed for identifing more than one genetic condition. Double homozygosity represented the causal mechanism in only half of them, whereas the other half showed peculiar multilocus combinations. The paper takes into consideration difficulties and learned lessons from our experience and therefore supports the powerful role of wide analyses for ascertaining multiple genetic diseases in complex patients, especially when a clinical suspicion could account for the majority of clinical signs. It finally makes clear how a patient’s “deep phenotyping” might not be sufficient to suggest the presence of multiple genetic diagnoses but remains essential to validate an unexpected multilocus result from genetic tests.

## 1. Introduction

Traditionally, in clinical genetic settings, identifying the correct diagnosis in a patient requires collecting all the history and physical hallmarks and then recognizing a pattern of a single known genetic condition that could explain all of them, in a “single-disorder” paradigm. The presence of additional clinical features that do not fit into the known pattern of the condition could either suggest a phenotype expansion, or an apparently new condition [1,2]. The finding of a pathogenic variant consistent with the majority of the patient’s clinical features usually stops any further genetic testing.

However, the expanding use of wide next-generation sequencing (NGS) analyses brought to evidence an unnegligible number of patients whose phenotype is caused by the association of multiple genetic conditions. The proportion of multiple disorders in a single patient has been estimated at approximately 2–7.5% of diagnosed cases, largely depending on the studied cohort [1,2,3,4]. In this scenario, single-gene tests or gene panels focused on a limited number of genes might be potentially inadequate, hiding possible adjunctive genetic variations based on an apparent phenotypic expansion.

Smith and colleagues indeed performed a retrospective review of multiple findings in diagnostic exome sequencing and observed that they were three times more frequent in patients from consanguineous families compared to patients from non-consanguineous families, due to co-inheritance of recessive disorders [2]. Even if more described, double homozygosity in autosomal recessive disease genes is not the unique inheritance mechanism for multiple diagnoses, as well as parental consanguinity isnot the only aspects to consider for suspecting multiple genetic defects in a patient. For example, in a large cohort of patients, Posey et al. found that the most commonly observed pattern of double diagnoses was two pathogenic variants in autosomal dominant disease genes [1].

Beyond large cohorts, a few single clinical reports in the literature described patients affected by comorbid conditions [5,6,7,8].

In this paper, we report the clinical and molecular characterization of eight representative patients for whom trio-based Whole Exome Sequencing (WES) allowed identifying more than one genetic condition with different inheritance patterns. The article thus highlights the role of WES in providing complete and fast diagnosis in patients with complex presentations of rare genetic syndromes, with important implications in the assessment of recurrence risk.

## 2. Materials and Methods

Over the last 7-year period (2015–2021), 2573 patients were referred to our laboratory to perform trio-WES analysis.

This study complied with the Declaration of Helsinki and was approved by the Ethics Committee of ASST Papa Giovanni XXIII of Bergamo as part of the RARE project (Rapid Analysis for Rapid Care) and the GENE Project (Genomic analysis Evaluation NEtwork).

After genetic counselling and written informed consent, genomic DNA was extracted from peripheral blood samples of probands and parents using standard procedures. The exonic regions and flanking splice junctions of the genome were captured using the Clinical Research Exome v2 kit (Agilent Technologies, Santa Clara, CA, USA). Sequencing was done on a NextSeq500 Illumina system with 150 bp paired-end reads. Reads were aligned to human genome build GRCh37/UCSC hg19, and analyzed for sequence variants using a custom-developed analysis tool [9]. The variant call file (vcf), including single nucleotide polymorphism and indels, was annotated by querying population frequencies databases and mutation databases, including the Genome Aggregation Database (http://gnomad.broadinstitute.org/, accessed on 19 June 2022), ClinVar (https://www.ncbi.nlm.nih.gov/clinvar/, accessed on 19 June 2022), and Human Gene Mutation Database Professional (HGMD, Release 2017.4). To prioritize variants, a sequential filtering strategy was applied, retaining only variants with the following characteristics: (a) potential effect on protein and transcript (splicing, missense, nonsense, and frameshift); (b) consistency with the patient’s phenotype according to the Human Phenotype Ontology classification (www.human-phenotype-ontology.org/, accessed on 19 June 2022); (c) consistency with the suspected inheritance model (autosomal recessive or de novo) with a frequency in the general population compatible with prevalence and incidence of the disease and showing a pathogenic mechanism corresponding to the one expected for the disease [9]. Variants were classified based on ACMG guidelines [10] (Supplementary Table S1). The potential causative variants were subsequently confirmed by Sanger sequencing in the proband and parents using an independent DNA sample.

Two pipelines were used to identify the copy number variants (CNVs) based on ExomeDepth and one created in-house, as previously described [11]. All the CNVs detected by both pipelines were annotated by matching every call with the genes involved and related diseases and classified according to ACMG and ClinGen guidelines [12].

## 3. Results

Among the diagnosed cases of our cohort, trio-WES allowed us to detect two independent genetic conditions in about 2.5% of them. Herein, we retrospectively described the clinical and molecular characterization of eight representative double-diagnosed patients.

### 3.1. Case 1

A 16-year-old boy, born from a non-consanguineous Italian couple, was referred to genetic evaluation for a polymalformative clinical picture characterized by mild hypoplastic cerebellum at brain magnetic resonance imaging (MRI), cleft palate, heptadactyly of the left hand, bilateral clubfoot with bilateral postaxial hexadactyly, agenesis of the right kidney, hypo-dysplasia of the left one (requiring renal transplantation at 4 years old), anal stenosis with ano-cutaneous fistula, and shawl scrotum. Karyotype analysis, performed right after birth, turned out normal (46, XY).

At the first genetic evaluation, at approximately 16 years old, his weight was at 75–90th centile, height was at 25–50th centile and head circumference was at 90th centile. Minor facial anomalies were observed, in particular small ears, bushy eyebrows, and a mildly short philtrum. He also presented brachydactyly with bilateral clinodactyly of the fifth finger, a proximal implant of both halluces and slightly shortened-appearing limbs. The patient had normal cognitive functions and was successfully attending secondary school.

An array-comparative genomic hybridization (aCGH), together with GLI3 analysis (NGS and Multiplex Ligation-dependent Probe Amplification) were carried out, with normal results.

At 18 years old, he underwent left hip joint replacement because of femoral head osteonecrosis, which was also present to a lesser degree on the contralateral leg.

Taking into consideration the non-specific clinical presentation, trio-based WES analysis was performed and detected two different homozygous variants. The first one was a missense pathogenic variant in *SLC26A2* (NM_000112.4:c.835C>T; p.Arg279Trp), previously reported in Recessive Multiple Epiphyseal Dysplasia type 4 (OMIM #226900) [13] and accounting for his cleft palate, short limb appearance, and hip dysplasia that probably led to femoral head necrosis. The other was a novel and likely pathogenic variant found in *IFT27* (NM_006860.4:c.350G>A; p.Gly117Asp), associated with Bardet-Biedl Syndrome 19 (OMIM #615996) [14,15]. Parents were heterozygous for both variants; the analysis also revealed multiple regions of genome-wide homozygosity in different chromosomes, suggesting a probable “territorial” consanguinity.

### 3.2. Case 2 and Case 3

The second patient was a girl born from two Tunisian second cousins. She was diagnosed at 5 years old with complete situs viscerum inversus (dextrocardia and abdominal organs) and progressive intrahepatic cholestasis, which led to liver cirrhosis. She underwent her first orthotopic liver transplant at 10 years old and required three subsequent re-grafts because of thrombotic complications. At the last evaluation (15 years old), her height and weight were under the 3rd centile and she presented a pubertal delay.

Her third-grade cousin (case 3) was a 2-year old girl affected by dextrocardia and mild psychomotor delay. Her little brother presented dextrocardia too. At approximately 1 year old, she was admitted to the hospital for persistent pruritus; an abdomen ultrasound revealed hepatosplenomegaly, whereas her blood tests showed severe cryptogenic hypertransaminasemia and elevated levels of bile acids, without evidence of infection by hepatotoxic viruses. An abdominal MRI confirmed complete situs viscerum inversus with polysplenia and hypertrophic hepatic lobes without nodular findings. At the evaluation, minor and non-peculiar facial anomalies were observed (epicanthal folds, pointed helixes, and saddle nose).

Trio-based WES analysis revealed in both patients two homozygous variants, respectively, in *DNAI1* and *TJP2* genes, both classified as likely pathogenic. The variant p.Gly651Glu, harbouring in the *DNAI1* gene (NM_012144.4:c.1952G>A), is associated with a form of Ciliary Dyskinesia with or without situs inversus (OMIM #244400); the second one, p.Gly532Arg, in the *TJP2* gene (NM_004817.4:c.1594G>A), accounts for progressive familial intrahepatic cholestasis (OMIM #615878).

### 3.3. Case 4

Genetic consultation was requested for a newborn born of Bangladeshi first-cousin parents that presented diffused skin macules with hyperchromic and hypochromic stains, variable in sizes and with jagged edges, some of which followed Blaschko lines; she also had multiple soft cafè-au-lait macules on the thoracic and dorsal regions. No freckling, dysmorphisms, or body asymmetries were detected.

To investigate the presence of Lisch nodules, she underwent appropriate ophthalmologic and fundus oculi examinations that showed no abnormalities.

After 5 months, she was admitted to the hospital for feeding difficulties, growth arrest, and worsening respiratory distress. Chest computed tomography (CT) showed severe bilateral pneumonia and progressively developed respiratory failure that required invasive ventilation. Karyotype on skin fibroblast was performed and results were normal (46, XX). Her blood tests revealed severe lymphopenia, agammaglobulinemia, and abnormal lymphocyte subpopulations (absence of T and B lymphocytes with normal natural killer cell count); these findings led to the suspicion of severe combined immunodeficiency. Because of the worsening of the clinical course, urgent trio-WES was performed on the peripheral blood and detected a homozygous pathogenic variant in *DCLRE1C* (NM_001033855.3:c.95C>G; p.Ser32Cys) [16,17], consistent with the diagnosis of severe combined immunodeficiency (OMIM #602450). Furthermore, her skin phenotype was explained by the identification of the novel homozygous likely pathogenic variant p.Met2158fs in the *ATM* gene (NM_000051.4:c.6472_6473del), responsible for Ataxia-telangiectasia syndrome (OMIM #208900). Parents were heterozygous carriers for each variant.

Thanks to immunoglobulin replacement and appropriate antimicrobial drug administration, she gradually improved and could be extubated.

### 3.4. Case 5

Trio-WES was performed on a 13-year-old girl for a neurological complex clinical picture.

Her epilepsy began at 4 years old with a generalised tonic-clonic seizure during sleep. Despite multiple anticonvulsive treatments, she continued having episodes of sudden loss of leg tone with consequent falls to the ground and multi-daily crisis of oral automatisms and hyperventilation, with suspected loss of contact. She also presented with a moderate intellectual disability, a dyskinetic movement disorder characterised by bradykinesia and stiffness, hypotonia, sleep disturbance, stereotypic movements, and feeding difficulties. Repeated brain MRIs showed minor aspecific anomalies and a lack of myelination in bilateral parieto-occipital lobes, whereas MR spectroscopy detected a markedly decreased N-acetylaspartate (NAA) signal of unknown explanation. At the genetic evaluation, she presented a long and narrow face, downslanting palpebral fissures, simplified ears, and constantly open mouth with thick gums and ogival palate. She also had slender and long limbs, hypotrophic thenar and hypothenar eminences, articular limitation of the knees and valgus feet. Her height was at 25–50th centile, weight at <3rd centile, and OFC at 50–75th centile; her arm span was proportionate.

There were previously carried out karyotype and fluorescence in situ hybridization (FISH) analyses for chromosome 22q11.2, aCGH, and an NGS panel of 28 genes related to Rett syndrome and differential diagnosis, all with normal results. Trio-WES identified a double genetic cause of her complex neurocognitive disorder. It was partly explained by a de novo likely pathogenic variant in the *GRIN2B* gene (NM_000834.5:c.1246T>C; p.Phe416Leu) which causes a dominant neurodevelopmental disorder (OMIM #616139) with described abnormal movement disorders such as dystonia or dyskinesia [18]. Moreover, WES identified two novel likely pathogenic variants in the *SLC25A12* gene, responsible for autosomal recessive epileptic encephalopathy, a paternally inherited missense variant p.Arg586Gln (NM_003705.5:c.1757G>A) combined with a frameshift variant p.Phe39fs (NM_003705.5:c.116_117del), occurring de novo on the maternal allele.

### 3.5. Case 6

A 3-year-and-8-month-old boy was referred to genetic evaluation for neurosensorial deafness and ichthyosis. He was born to consanguineous parents (first-grade cousins) from Pakistan; both parents and two brothers were normal-hearing.

He presented with normal motor development, but no language due to severe profound sensorineural hearing loss on all frequencies. A brain MRI and a CT showed regular cochleovestibular apparatus; therefore, he underwent a right cochlear implant. Eye examination and fundus oculi results were normal.

He had normal stature-ponderal growth, absence of face dysmorphisms or anomalies of ear auricles. Dermatologists had already visited the child, making a diagnosis of ichthyosis, with dark brownish scales, more evident on the abdomen, arms, and legs; his palms and soles were lesion-free.

Supposing a single genetic cause for his clinical picture, trio-WES was performed and unexpectedly resulted in two conditions; in fact, it highlighted two variants of unknown significance, the paternal p.Arg202Gln (NM_144672.4:c.605G>A) and the maternal p.Thr322Ile (NM_144672.4:c.965C>T) in the *OTOA* gene, whose biallelic mutations underlie moderate to severe prelingual sensorineural deafness (OMIM #607039). The simultaneous use of CNV-detection tool led to the identification of a microdeletion on the short arm of the X chromosome, maternally inherited, of approximately 928 kb (ChrX:6966861-7895483, GRCh37/hg19) that included the *STS* gene. Deletions of STS are implicated in 80–90% of cases affected by X-linked ichthyosis (OMIM #308100) [19].

### 3.6. Case 7

A girl with a previous diagnosis of Prader-Willi syndrome (OMIM #176270) was referred for trio-WES analysis.

She was the third child of non-consanguineous parents; her family history was unremarkable. Pregnancy was uneventful except for growth restriction at the last ultrasound. Soon after birth, she presented with severe hypotonia and feeding difficulties for which she required enteral tube feeding. The clinical suspect of Prader-Willi syndrome (PWS) was confirmed by detection of the maternal uniparental disomy (UPD) of the critical region 15q11.2-q13.

From 2 and a half years old, after achieving walking alone and speaking, she presented an abrupt arrest in motor and verbal acquisitions followed by a constant regression of motor functions until she gradually lost her walking ability and standing position and developed unmotivated laughing episodes associated with loss of axial and head control and head-nodding stereotypes. At approximately 5 years of age, following an episode of gastroenteritis, she presented a rapid worsening of motor skills, with increased hypotonia, reflexes and muscle weakness, no autonomous feeding nor sphincter control. Her height was at the 10th centile and her weight was under the 3rd centile for age.

An electroencephalogram detected focal anomalies on the bilateral posterior regions and diffused sequence waves of an uncertain nature. A brain MRI showed cerebral and cerebellar atrophy, hypomyelination, particularly of periventricular zones and abnormal signals on the basal ganglia.

On suspicion of a neurodegenerative disorder in addition to her PWS diagnosis, an urgent trio-WES was performed and detected the homozygous pathogenic variant p.Tyr142fs (NM_017882.3:c.424dup) in the *CLN6* gene, consistent with an infantile form of neuronal ceroid lipofuscinosis (OMIM #601708). The variant was inherited only from the mother; its homozygosity was explained by the localisation of the *CLN6* gene on chromosome 15q23, inside the region of maternal uniparental disomy.

### 3.7. Case 8

A 16-year-old male with neonatally diagnosed Williams-Beuren syndrome (WBS) (OMIM #194050) was sent for trio-WES analysis for supposed double comorbidity. Born from unrelated Italian parents, his family history was significant, because his father, paternal grandfather, and grandfather’s brother all presented with congenital anosmia.

In the first months of life, WBS was diagnosed based on a large perimembranous ventricular septal defect with mild supravalvar aortic stenosis, developmental delay, feeding difficulties with poor weight gain, and distinctive facies. An array-CGH resulted indeed in a de novo 7q11.23 microdeletion of about 1.4 Mb.

From 3 years old, he also developed hypothyroidism, mild myopia, and important language regression. Focal epilepsy arose at approximately 12 years old, which was resistant to combined anticonvulsive therapy, and a subsequent brain MRI revealed a lesion with an altered signal at the paramedian pontine zone of uncertain origin and hypoplasia of the olfactory tracts. Nevertheless, his condition gradually evolved into a severe intellectual disability, with language regression and scoliosis.

At the last auxological evaluation, his weight and height were at −2 DS on specific WBS growth curves [20].

Concerning his complex neurocognitive picture, not fully explainable by his WBS diagnosis, trio-WES was performed and a de novo heterozygous likely pathogenic variant was identified in *TNPO2*, p.Arg105ter (NM_001136196.2:c.313C>T), consistent with a concurrent novel neurodevelopmental disorder (OMIM #619556) [21].

## 4. Discussion

The reported double-diagnosed cases are a good illustration, on top of previously described ones [5,6,7,8], of how different genetic conditions and molecular mechanisms could combine in a single patient, causing peculiar and complex clinical pictures. In Table 1, we summarised the combined diagnoses and their contribution to the patient’s phenotype of our eight cases together with two other cases previously published by Cianci et al. [5] and Pezzani et al. [7] and diagnosed in our laboratory as well.

According to what was reported by Smith and colleagues [2], the more prevalent causing mechanism, in our cohort, was double homozygosity (cases 1 to 4), especially for children of consanguineous parents. Nevertheless, it was not the only one; our fifth case presented a de novo mutation in a dominant gene, along with a defective recessive one, because of a paternally inherited mutation and a de novo variant on the other allele. The sixth case, in spite of parental consanguinity, showed compounded heterozygous variants in a recessive gene, combined with a maternal pathogenic microdeletion revealed by WES. The seventh case was an already diagnosed maternal UPD that concurrently unmasked a pathogenic maternal variant in an included gene. The last case was affected by two de novo diseases: a recurrent microdeletion and an additional dominant *TNPO2*-neurodevelopmental disorder; interestingly, only 15 other patients carrying de novo pathogenic variants in *TNPO2* have been reported to date [21].

The natural tendency in medical genetics has always been to find a diagnosis that could explain all the patient’s characteristics, in a “single-disorder” paradigm; in doing so, the non-classical signs relatable to the detected diagnoses were mainly classified as “phenotypic expansions” or “atypical presentations” [4]. However, in the last few years, it became evident that a significant part of so-called atypical cases actually represented “blended” (i.e., mixed phenotypes with overlapping features) or “composite” (i.e., distinct phenotypes that singularly explain patient’s characteristic) cases with multiple genetic conditions.

For clinicians, supposing comorbid conditions could be more straightforward when the patient has multiple features that do not fit one single diagnosis, when his clinical picture is more severe than expected by the first diagnosis, or when there are only some signs that independently segregate in the family, especially with a history of consanguineous parents [2,3]. For example, in our last case, a comorbid condition besides the WBS diagnosis was easily suspected because of the unusual epileptic encephalopathy together with a severe speech impairment; moreover, the patient’s hypoplasia of the olfactory tracts and the congenital anosmia that was segregated in his paternal family suggests a possible third unrevealed condition.

On the other hand, presuming multiple diagnoses is much more challenging when the conditions are overlapping, i.e., when one or more features could be attributable to both diseases [2]; and it is even more arduous when the overlapping features are the main clinical signs of both diseases (as in our cases 4 and 5). Another tricky situation, rarely discussed in precedent reports, is when multiple conditions, despite having distinct phenotypes, deviate the suspect towards a third disease that includes all the patient’s features; a representative case is our sixth patient in which the initial suspicion was a “Keratitis-ichthyosis-deafness syndrome” whereas trio-WES returned two independent genetic causes.

Taking into account the above, the probability of correctly diagnosing multiple genetic conditions strictly depends on the patient’s “deep phenotyping” and on what type of genetic tests are used in the diagnostic journey. Concerning this, it seems evident that wide analyses, such as whole-exome and whole-genome sequencing, are the most powerful tools to bring to light multiple conditions in one single patient. This could be even more appropriate thanks to the ongoing incorporation of technologies for copy-number variants (CNVs) detection in the WES pipeline, uncovering at the same time monogenic and genomic disorders [22], as in Case 6. For example, copy number variants and single-nucleotide variants as a part of multiple diagnoses were reported in 11.9% of double-diagnosed patients by Posey et al. [1], whereas Chen et al. recently applied simultaneous CNV-seq and WES analysis on a large cohort of malformed fetuses and detected coincidental pathogenic CNVs and single gene variants in about 1% of them. In some of these cases, using the standard workflow of sequential karyotype, aCGH and WES, might lead to a premature halt in the diagnostic path [23].

Striving to find the complete diagnosis in genetic patients is essential for tailoring the correct management and follow-up. It also has strong implications for the rest of the family, particularly for the right assessment of parents’ reproductive risk and for providing correct prenatal options; the two conditions can in fact segregate independently or they could be linked to each other, as in the case 7. Furthermore, in a few rare cases, it could lead to an important rebound for the parents’ health itself, as with the mother of case 4 in that being a heterozygous carrier of an ATM mutation has to undergo stricter breast cancer screening [24].

## 5. Conclusions

The report illustrates how different co-occurring phenotypes and inheritance patterns might cause blended or composite clinical pictures, sometimes in a misleading and challenging way for clinicians. In this scenario, the patient’s “deep phenotyping” might not be enough to suggest the presence of multiple genetic diagnoses; however, it remains essential to validate an unexpected multilocus result from genetic tests.

As stated by several studies, our cases further support the increasing evidence of how the adoption of genome-wide sequencing analyses (such as trio-WES) as first-tier sequencing tests can ensure accurate and time-saving diagnosis, particularly for complex patients [25,26].

## Figures and Tables

**Table 1:** Overview of the patients’ genetic diagnosis and their respective contributions to the phenotypes.

Case	Sex	Age at Diagnosis	Parental Consanguinity	1st Diagnosis	1st Molecular Mechanism	Features Caused by 1st Diagnosis	2nd Diagnosis	2nd Molecular Mechanism	Features Caused by 2nd Diagnosis	Phenotypic Overlap	Additional Features
**CASE 1**	M	20 years 1 month	Not known, probably “territorial”	Multiple epiphyseal dysplasia, type 4 OMIM #226900 AR	*SLC26A2* hmz p.Arg279Trp, mat/pat	Cleft palate, bilateral osteonecrosis of femoral head, short limbs	Bardet-Biedl syndrome 19 OMIM #615996 AR	*IFT27* hmz p.Gly117Asp, mat/pat	Relative macrocephaly, cerebellar hypoplasia, kidney disease, post-axial polydactyly, anal stenosis	Brachyclynodactyly, bilateral clubfeet	Shawl scrotum
**CASE 2**	F	15 years 6 months	Yes (second cousins)	Ciliary dyskinesia, primary, 1, with or without situs inversus OMIM #244400 AR	*DNAI1* hmz p.Gly651Glu, mat/pat	Complete situs viscerum inversus	Cholestasis, progressive familial intrahepatic 4 OMIM #615878 AR	*TJP2* hmz p.Gly532Arg, mat/pat	Progressive cholestasis, liver cirrhosis, poor growth	/	Pubertal delay
**CASE 3**	F	2 years 8 months	Not reported	Ciliary dyskinesia, primary, 1, with or without situs inversus OMIM #244400 AR	*DNAI1* hmz p.Gly651Glu, mat/pat	Complete situs viscerum inversus and polysplenia	Cholestasis, progressive familial intrahepatic 4 OMIM #615878 AR	*TJP2* hmz p.Gly532Arg, mat/pat	Persistent pruritus, hepatosplenomegaly, increased serum bile acids, hypertransaminasemia	/	Mild psychomotor delay and minor facial anomalies
**CASE 4**	F	7 months	Yes (first cousins)	Severe combined immunodeficiency, Athabascan type OMIM #602450 AR	*DCLRE1C* hmz p.Ser32Cys, mat/pat	Failure to thrive, decreased numbers of B cells, agammaglobulinemia	Ataxia-teleangectasia OMIM #208900 AR	*ATM* hmz p.Met2158fs, mat/pat	Cafè-au-lait spots, hypo-/hyperpigmented macules	Immunodeficiency	/
**CASE 5**	F	14 years 4 months	No	Intellectual developmental disorder, with or without seizures OMIM #613970 #616139 AD	*GRIN2B* htz p.Phe416Leu, de novo	Dyskinetic movement disorder, autistic features and feeding difficulties	Developmental and epileptic encephalopathy OMIM #612949 AR	*SLC25A12* htz p.Arg586Gln, pat htz p.Phe39fs, de novo	Lack of brain myelination, decreased N-acetyl aspartate on MR spectroscopy	Intellectual disability, epilepsy, hypotonia	Aspecific minor facial anomalies
**CASE 6**	M	4 years 9 months	Yes (first cousins)	Deafness, autosomal recessive 22 OMIM #607039 AR	*OTOA* htz p.Arg202Gln, pat htz p.Thr322Ile, mat	Sensorineural severe deafness	Ichthyosis OMIM #308100 X-linked recessive	*STS* Xp22.31 deletion, mat	Ichthyosis	/	/
**CASE 7**	F	Right after birth (1st) and 5 years 9 months (2nd)	No	Prader-Willi syndrome OMIM #176270 Imprinting disorder	Maternal UPD of 15q11.2-23 region *	Severe hypotonia, feeding difficulties, global developmental delay	Ceroid lipofuscinosis, neuronal, type 6A OMIM #601708 AR	*CLN6* hmz p.Tyr142fs, mat	Progressive cognitive decline, loss of motor and language skills, cerebral and cerebellar atrophy, hypomyelination, EEG anomalies	/	/
**CASE 8**	M	2 months (1st) and 16 years old (2nd)	No	Williams-Beuren syndrome OMIM #194050 Genomic disorder	7q11.23 microdeletion, de novo *	Perimembranous VSD, supravalvular aortic stenosis, distinctive facies, hypothyroidism, scoliosis	Intellectual developmental disorder with hypotonia, impaired speech, and dysmorphic facies OMIM #619556 AD	*TNPO2* htz p.Arg105ter, de novo	Epilepsy, EEG abnormalities, developmental regression, important speech impairment, MRI brain abnormalities	Intellectual disability, feeding difficulties, poor overall growth, myopia	Hypoplasia of the olfactory tracts
**Cianci et al., 2019** **[5]**	F	2 years and 6 months (1st) and 4 years and 7 months (2nd)	No	Neurofibromatosis, type 1 OMIM #162200 AD	*NF1* htz p.Lys2401fs, mat *	Café-au-lait spots, groin and axillary freckling, UBOs at brain MRI	KBG syndrome OMIM #148050 AD	*ANKRD11* htz p.Phe904fs, de novo	Postnatal short stature, moderate intellectual disability, facial dysmorphisms, macrodontia of upper central incisors	/	/
**Pezzani et al., 2019** **[7]**	M	4 months	Yes (first cousins)	Mosaic variegated aneuploidy syndrome 2 OMIM #614114 AR	*CEP57* hmz p.Leu309Profs*9, mat/pat	IUGR, congenital hypothyroidism, congenital heart defects	Short-rib thoracic dysplasia 3 with or without polydactyly OMIM #613091 AR	*DYNC2H1* hmz p.Met3762Val, mat/pat	Polydactyly, brachydactyly, cystic liver, recurrent respiratory infections	Rhizomelic shortening of the limbs and bell-shaped thorax	Butterfly vertebra, supernumerary rib, recurrent infections and immunodeficiency, vascular malformations

* Not-detected by trio-WES. AD: autosomal dominant; AR: autosomal recessive; EEG: electroencephalogram; hmz: homozygous; htz: heterozygous; IUGR: intrauterine growth restriction; mat: maternal; MR: magnetic resonance; MRI: magnetic resonance imaging; pat: paternal; UBO: udinditified bright object; UPD: uniparental disomy; VSD: ventricular septal defect.

## Data Availability

The WES data supporting the findings of this study are available on request from the last author (M.I.). The data are not publicly available due to privacy/ethical restrictions.

## Supplementary Materials

The following supporting information can be downloaded at: https://www.mdpi.com/article/10.3390/genes13071275/s1, Supplementary Table S1: Supporting criteria for classification of variants identified with trio-WES in our cases [27,28].
