# Supplementary material for: Atypical, Composite, or Blended Phenotypes: How Different Molecular Mechanisms Could Associate in Double-Diagnosed Patients
Source: Genes (Basel). 2022 Jul 19;13(7):1275. doi: 10.3390/genes13071275 (PMC9319862; doi:10.3390/genes13071275)
Supplement: Supplementary file 1 [file genes-13-01275-s001.zip › genes-1802959-supplementary.pdf]

Supplementary Table S1. Supporting criteria for classification of variants identified with trio-WES in our cases.

Case	Gene	Variant	Heterozygosity	GnomAD MAF	ClinVar	Literature	ACMG criteria [10]	ACMG classification [10]
**Case 1**	*SLC26A2* OMIM *606718	NM_000112.4 c.835C>T p.Arg279Trp	homozygous	271/282628	Pathogenic	Hastbacka et al.(1996) [27]	PS1;PS3;PM1;PP3;PP5	Pathogenic
	IFT27 OMIM *615870	NM_006860.4 c.350G>A p.Gly117Asp	homozygous	0	NA	NA	PM1;PM2;PP2;PP3	Likely pathogenic
**Case 2 and 3**	*DNAI1* OMIM *604366	NM_012144.4 c.1952G>A p.Gly651Glu	homozygous	0	NA	NA	PM1;PM2;PP3;PP4	Likely pathogenic
	TJP2 OMIM *607709	NM_004817.4 c.1594G>A p.Gly532Arg	homozygous	2/1211386	Pathogenic	NA	PM1;PM2;PP3;PP5	Likely pathogenic
**Case 4**	*DCLRE1C* OMIM *605988	NM_001033855.3 c.95C>G p.Ser32Cys	homozygous	1/250754	NA	Le Deist et al.(2004) [16]	PS1;PS3;PM1;PM2;PP 3	Pathogenic
	ATM OMIM *607585	NM_000051.4 c.6472_6473del p.Met2158fs	homozygous	0	NA	NA	PVS1;PM2	Likely pathogenic
**Case 5**	*GRIN2B* OMIM *138252	NM_000834.5 c.1246T>C p.Phe416Leu	heterozygous, de novo	0	NA	NA	PS2;PM2;PP3;BP4	Likely pathogenic
	SLC25A12 OMIM *603667	NM_003705.5 c.116_117del p.Phe39fs	heterozygous, de novo (maternal allele)	0	NA	NA	PS2;PM2	Likely pathogenic

		NM_003705.5 c.1757G>A p.Arg586Gln	heterozygous, paternal	2/282776	NA	NA	PM1;PM2;PM3;PP3	Likely pathogenic
**Case 6**	*OTOA* OMIM *607038	NM_144672.4 c.605G>A p.Arg202Gln	heterozygous, paternal	7/251128	NA	NA	PM2;BP4	VUS
		NM_144672.4 c.965C>T p.Thr322Ile	heterozygous, maternal	0	NA	NA	PM2;BP4	VUS
**Case 7**	*CLN6* OMIM *606725	NM_017882.3 c.424dup p.Tyr142fs	homozygous	0	NA	Berkovic et al.(2019) [28]	PVS1;PS4;PM2	Pathogenic
**Case 8**	*TNPO2* OMIM *603002	NM_001136196.2 c.313C>T p.Arg105ter	heterozygous, de novo	0	NA	NA	PS2;PM2	Likely pathogenic

Abbreviations: BP: benign supporting; MAF: minor allele frequency; NA: not available; PM: pathogenic moderate; PP: pathogenic supporting; PS: pathogenic strong; PVS: pathogenic very strong; VUS: variant of unknown significance.

References

10. Richards, S.; Aziz, N.; Bale, S.; Bick, D.; Das, S.; Gastier-Foster, J.; Grody, W.W.; Hegde, M.; Lyon, E.; Spector, E.; et al. Standards and guidelines for the interpretation of sequence variants: A joint consensus recommendation of the American College of Medical Genetics and Genomics and the Association for Molecular Pathology. *Genet. Med*. **2015**, *17*, 405–424. https://doi.org/10.1038/gim.2015.30.

16. Le Deist, F.; Poinsignon, C.; Moshous, D.; Fischer, A.; de Villartay, J.P. Artemis sheds new light on V(D)J recombination. *Immunol. Rev*. **2004**, *200*, 142–155. https://doi.org/10.1111/j.0105-2896.2004.00169.x.

27. Hästbacka, J.; Superti-Furga, A.; Wilcox, W.R.; Rimoin, D.L.; Cohn, D.H.; Lander, E.S. Atelosteogenesis type II is caused by mutations in the diastrophic dysplasia sulfate-transporter gene (DTDST): evidence for a phenotypic series involving three chondrodysplasias. *Am. J. Hum. Genet*. **1996**, *58*, 255-62.

28. Berkovic, S.F.; Oliver, K.L.; Canafoglia, L.; Krieger, P.; Damiano, J.A.; Hildebrand, M.S.; Morbin, M.; Vears, D.F.; Sofia, V.; Giuliano, L.; et al. Kufs disease due to mutation of CLN6: clinical, pathological and molecular genetic features. *Brain* **2019**, *142*, 59-69. https://doi: 10.1093/brain/awy297.
